# Innate Immune Responses of *Galleria mellonella* to *Mycobacterium bovis* BCG Challenge Identified Using Proteomic and Molecular Approaches

**DOI:** 10.3389/fcimb.2021.619981

**Published:** 2021-02-09

**Authors:** Masanori Asai, Gerard Sheehan, Yanwen Li, Brian D. Robertson, Kevin Kavanagh, Paul R. Langford, Sandra M. Newton

**Affiliations:** ^1^ Section of Paediatric Infectious Disease, Department of Infectious Disease, Imperial College London, London, United Kingdom; ^2^ SSPC Pharma Research Centre, Department of Biology, Maynooth University, Maynooth, Ireland; ^3^ Institute of Microbiology and Infection, School of Biosciences, University of Birmingham, Birmingham, United Kingdom; ^4^ MRC Centre for Molecular Bacteriology and Infection, Department of Infectious Disease, Imperial College London, London, United Kingdom

**Keywords:** *Galleria mellonella*, *Mycobacterium bovis* BCG, tuberculosis, innate immunity, *in vivo* model, proteomics, gene expression

## Abstract

The larvae of the insect *Galleria mellonella*, have recently been established as a non-mammalian infection model for the *Mycobacterium tuberculosis* complex (MTBC). To gain further insight into the potential of this model, we applied proteomic (label-free quantification) and transcriptomic (gene expression) approaches to characterise the innate immune response of *G. mellonella* to infection with *Mycobacterium bovis* BCG *lux* over a 168 h time course. Proteomic analysis of the haemolymph from infected larvae revealed distinct changes in the proteome at all time points (4, 48, 168 h). Reverse transcriptase quantitative PCR confirmed induction of five genes (*gloverin*, *cecropin*, *IMPI*, *hemolin*, and *Hdd11*), which encoded proteins found to be differentially abundant from the proteomic analysis. However, the trend between gene expression and protein abundance were largely inconsistent (20%). Overall, the data are in agreement with previous phenotypic observations such as haemocyte internalization of mycobacterial bacilli (hemolin/β-actin), formation of granuloma-like structures (Hdd11), and melanization (phenoloxidase activating enzyme 3 and serpins). Furthermore, similarities in immune expression in *G. mellonella*, mouse, zebrafish and *in vitro* cell-line models of tuberculosis infection were also identified for the mechanism of phagocytosis (β-actin). Cecropins (antimicrobial peptides), which share the same α-helical motif as a highly potent peptide expressed in humans (h-CAP-18), were induced in *G. mellonella* in response to infection, giving insight into a potential starting point for novel antimycobacterial agents. We believe that these novel insights into the innate immune response further contribute to the validation of this cost-effective and ethically acceptable insect model to study members of the MTBC.

## Introduction

Tuberculosis (TB) is the leading cause of global infectious disease mortality, with a quarter of the world’s population believed to be infected with the causative agent, *Mycobacterium tuberculosis* (MTB) (World Health Organization [WHO], 2020). A large majority of this population will live with a non-contagious and asymptomatic infection known as latent TB infection. Amongst these individuals, 5%–10% will develop active TB disease over their lifetime, serving as a reservoir for future transmission and infection (WHO, 2020). WHO is currently working to end the global TB epidemic by 2035. The first milestone goal is set for 2020, which will be missed by substantial margins (Reid et al., 2019). In order to meet the next milestone goal in 2035, significant advancement and increase in TB research output is needed to further understand the disease, and to identify novel mechanisms, drug targets and biomarkers, and the subsequent development of much needed tools to end the TB epidemic.

Animal infection models (e.g., mice, guinea pigs, macaques) have played an essential role in our current understanding of TB disease (Williams and Orme, 2016; Zhan et al., 2017). There are a wide variety of animal models used for TB research, but each comes with their limitations (Zhan et al., 2017). For example, in the most widely used mouse (C57Bl/6 and BALB/c) models, necrotic granulomas, a hallmark of TB, do not form (Orme, 2003). However, the ability to comprehensively study the immune response to infection, makes them useful despite their limitations. Furthermore, the use of animals is resource intensive, time consuming, requires the use of specialized animal facilities and is associated with ethical constraints, leading to a bottleneck in the research pipeline (Williams and Orme, 2016; Zhan et al., 2017). Over the past decade, the larvae of the insect, *Galleria mellonella* (Greater wax moth) have become increasingly popular as an infection model to study bacterial [e.g., *Klebsiella pneumoniae* (Insua et al., 2013), *Escherichia coli* (Alghoribi et al., 2014), *Staphylococcus aureus* (Sheehan et al., 2019)] and fungal [e.g., *Candida albicans* (Kelly and Kavanagh, 2011), *Aspergillus fumigatus* (Sheehan et al., 2018a) and *Madurella mycetomatis* (Sheehan et al., 2020b)] pathogens. Additionally the use of non-mammalian models, such as *G. mellonella*, in research is driven by the movement to reduce, replace and refine (3Rs) the use of vertebrate animals in scientific experimentation (Graham and Prescott, 2015). This insect model offers a number of advantages over conventional mammalian models: 1) low acquisition and maintenance costs, 2) accessibility, requiring minimal training and without the need for specialized equipment and facilities, 3) can tolerate incubation at 37°C, 4) no ethical constraints, 5) study of innate immunity in isolation from adaptive immunity, and 6) rapid infection cycle which allows for mid to high throughput data generation (Asai et al., 2019a). Furthermore pre-existing, non-mammalian infection models such as *Drosophila melanogaster* (fruit flies) and *Danio rerio* (zebrafish), are often incompatible with human pathogens, and require specialized facilities and equipment for infection and maintenance (Avdesh et al., 2012; Siva-Jothy et al., 2018).

Although the invertebrate *G. mellonella* lacks adaptive immunity, its innate immune system is complex and shares functional and anatomical similarities to those found in mammals (Wojda, 2017). The innate immune response of *G. mellonella* is broadly differentiated into two categories, cellular and humoral responses (Hillyer, 2016). The cellular response is driven by innate immune cells known as haemocytes (analogous to blood cells) (Pereira et al., 2018). At least six types of haemocytes have been identified in *G. mellonella*, with plasmatocytes and granulocytes being the most common types and the primary drivers of cellular functions such as phagocytosis, nodulation and encapsulation (Pereira et al., 2018). The humoral response is activated upon the detection of invading pathogens, through recognition of pathogen/damage associated molecular patterns (PAMPs/DAMPs) using pattern recognition receptors (PRRs), e.g., Toll-like receptors, β-1,3-glucan and IL-1R, and inducing signalling cascades, e.g., IMD, JNK, JAK-STAT pathways (Sheehan et al., 2018b). This leads to the expression and production of antimicrobial peptides (AMPs), mainly within the fat body (liver-like tissue), NADPH oxidase complex dependent production of reactive oxygen/nitrogen species (ROS/RNS) and hydrogen peroxide, which are secreted into the haemolymph found in the haemocoel (analogous to blood/blood vessel) (Sheehan et al., 2018b). The phenoloxidase (PO) cascade is responsible for modulation of melanization, which is a key process to localize and control infection through melanin deposition and production of phenolic compounds (Hillyer, 2016). The PO cascade system shares similarities with the mammalian complement cascade, e.g., both are initiated in response to pathogen recognition and are tightly regulated by proteases and protease inhibitors. However, they do differ in their final stages, i.e., melanization in insects and induction of cell lysis in mammals (Sheehan et al., 2018b).

Studies have demonstrated that *G. mellonella* is a viable host for mycobacterial species including both members of the MTB complex (MTBC), including MTB (Li et al., 2018; Asai et al., 2019a; Asai et al., 2019b; Asai et al., 2020), and non-tuberculous mycobacteria (NTM) (Entwistle and Coote, 2018; Meir et al., 2018; Kirubakar et al., 2020). Previously we established a *G. mellonella* – *Mycobacterium bovis* BCG *lux* model, characterised by establishment of intracellular and non-replicative but persistent infection, development of granuloma-like structures within the infected larvae, and a shift to a non-replicative lipid-rich BCG phenotype (Li et al., 2018). In addition, we demonstrated its capability as a rapid drug screen for antimycobacterial compounds, giving results within 96 h following infection (Asai et al., 2019b). We have employed BCG as a surrogate for MTB, due to its compliance with containment level (CL)/biosafety level (BSL) two facilities, thereby enabling researchers lacking specialized facilities (CL3/BSL3) to undertake TB research. While other surrogate mycobacteria exist, such as *Mycobacterium smegmatis*, BCG is considered to be superior due to its genetic similarity (>99.9% vs 70%) with MTB (Altaf et al., 2010). Furthermore, BCG has widely been used for *in vitro*/*ex vivo* mycobacterial growth inhibition assays (MGIAs) to study the immune mechanisms of mycobacterial control, proving to be a valuable organism in mycobacterial research (Roy et al., 2019; Tanner et al., 2019; Painter et al., 2020). Methodologies such as histopathology, transmission electron microscopy, bioluminescence, *ex vivo* haemocyte assays, and larval survival studies have typically been used to study the innate immune response to mycobacterial infection in *G. mellonella* (Li et al., 2018; Asai et al., 2019a; Asai et al., 2019b; Sheehan et al., 2019). However, greater knowledge of the larval innate immune system in response to mycobacterial infection is needed to further characterise and expand its potential as a model organism. In this study, and for the first time to our knowledge, we present a proteomic and reverse transcriptase quantitative PCR (RT-qPCR) analysis of the innate immune response of *G. mellonella* to infection with a member of the MTBC over a time course of 168 h.

## Methods

### Mycobacteria Culture Conditions

*M. bovis* BCG *lux*, is a genetically modified bioluminescent Montréal vaccine strain which has been transformed with the shuttle plasmid vector pSMT1 expressing the *luxAB* genes of *Vibrio harveyi* (Snewin et al., 1999; Newton et al., 2011). BCG *lux* was cultured in Middlebrook 7H9 media (BD Difco, UK) supplemented with 0.2% glycerol (Sigma-Aldrich, UK), 0.05% polysorbate-80 (Sigma-Aldrich, UK) and 10% albumin dextrose catalase (BD Difco, UK), 50 µg/ml of hygromycin (Roche, UK), and incubated at 37°C in an orbital shaker. For enumeration of colony forming units (CFU), 10-fold serial dilutions of BCG *lux* were plated onto Middlebrook 7H11 agar (BD Difco, UK) supplemented with 0.5% glycerol, 10% oleic acid albumin dextrose catalase (BD Difco, UK), 50 µg/ml of hygromycin, and statically incubated at 37°C. The bioluminescence, relative light unit (RLU)/ml of BCG *lux*, was measured using a luminometer (Berthold Technologies, DE) and 1% decanal (Sigma-Aldrich) as the substrate. RLU was used as a relative and rapid estimation of CFU at a ratio of 3:1/4:1 *in vitro* and *in vivo*, respectively, as previously determined (Li et al., 2018).

### *G. mellonella* Maintenance

Last instar *G. mellonella* larvae were purchased from Livefoods Direct, UK. Upon arrival, larvae were examined, and melanized or dead larvae were removed prior to storage in the dark at 18°C. Larvae were used within a week of arrival and were not fed during this period or throughout the experiment. Healthy larvae of approximately equal size (2–3 cm), weight (250 mg), motility and colour were picked for experimentation and acclimatised to room temperature before experimentation.

### *G. mellonella* Infection With BCG *lux*

Mid-log phase BCG *lux* was prepared and injection of healthy *G. mellonella* larvae were carried out as previously described (Asai et al., 2019a). *G. mellonella* larvae were injected with 10 µl of BCG *lux* inoculum (1 x 10^5^, 1 x 10^7^ or 2 x 10^7^ CFU) *via* the last left proleg of the larva. Injection is the preferred method over feeding, as dosing of BCG *lux* can be more accurately controlled. Infected larvae were transferred to a Petri dish lined with filter paper and incubated in a vented box at 37°C in the dark. N = 30 larvae were infected for each experimental group, unless otherwise stated, and each experiment consisted of three biological replicates (N = 90). The negative infection controls were naïve larvae, mock infected with phosphate-buffered saline (PBS), with tween (0.05%, PBS-T). Survival of the infected larvae were recorded for up to 1-week post infection (pi). Larvae were considered dead when they failed to respond to touch. Where appropriate, a t-test was carried out to determine statistical significance.

### Determination of BCG *lux* Load in *G. mellonella*

The survival of BCG *lux in vivo*, over a one week-time course was determined at 0, 24, 48, 72, 96, and 168 h pi. Five larvae were individually homogenized and the RLU of the homogenate was measured using a luminometer as previously described (Asai et al., 2019a). Background bioluminescence of the homogenate was previously reported as approximately 5,000 RLU/ml.

### Preparation of Larval Haemolymph for Proteomic Analysis

Collection of haemolymph for proteomic analysis was carried out using our previously established protocol (Sheehan and Kavanagh, 2018; Sheehan et al., 2018a; Sheehan et al., 2019; Sheehan et al., 2020b). Briefly, larval haemolymph was collected at 4, 48, and 168 h pi for proteomic analysis. Larvae were punctured between the head and the thorax using a sterile 30-gauge needle. Haemolymph from 10 larvae was pooled (three drops [approximately 60 μl]/larva) into a sterile 1.5 ml reaction tube containing a few pellets of N-phenylthiourea on ice to prevent melanization. The haemolymph was centrifuged at 10,000 x g for 10 min to pellet the cells, and 30 µl of the cell free haemolymph was transferred into a fresh 1.5 ml reaction tube containing 270 µl of PBS. Three independent samples were collected. Haemolymph from uninfected naive larvae was collected as a reference control.

### Label-Free Quantification (LFQ) Proteomics of Larval Haemolymph

Cell free haemolymph proteins (75 µg) were processed for proteomics using previously described and established protocols (Sheehan and Kavanagh, 2018; Sheehan et al., 2018a; Sheehan et al., 2019; Sheehan et al., 2020b). Protein identification from MS/MS data was performed using the Andromeda search engine in MaxQuant (v 1.2.2.5; http://maxquant.org/), correlating the data against the six-frame translation of the expressed sequence tags (EST) contigs for *G. mellonella*, with the addition of the proteomes of insect species *Hyalophora cecropia, Manduca sexta, Bombyx mori* (Vogel et al., 2011), and a proteomic database of *M. bovis* BCG (Brosch et al., 2007). The MS proteomic data and MaxQuant search output files were deposited to the ProteomeXchange Consortium *via* PRIDE partner repository with the dataset identifier PXD015250.

Data processing, statistical analysis, and graph generation were conducted using Persus (v 1.5.5.3). LFQ intensities were log_2_ transformed and one-way analysis of variance (ANOVA) and t-tests were carried out between the naïve reference and 4, 48, and 168 h BCG *lux* pi larval haemolymph samples. Significance was determined using p-value of 0.05 as a cut-off and Benjamini-Hochberg correction (Benjamini and Hochberg, 1995) was applied for false discovery rate. Proteins with non-existent values (absence or low abundance) were expressed as the imputation of the zero-value using a number close to the lowest value of the range of proteins plus or minus the standard deviation. Once imputed, these proteins were included in the analysis of total differentially expressed groups.

### Reverse Transcriptase Quantitative PCR (RT-qPCR)

To quantify gene expression in *G. mellonella* larvae infected with BCG *lux*, five larvae were individually homogenized using a Ribolyser in a 2 ml lysing tube containing 900 µl of TRIzol (Thermo Fisher Scientific, UK) and six 1/8-inch metal beads (MP Biomedical, USA) at 4, 48 and 168 h pi. The lysate was processed to extract RNA using an RNeasy Mini Kit (Qiagen, UK) following the manufacturer’s instructions. RNA of five larvae were pooled and cDNA was synthesised using the QuantiTect Reverse Transcription kit (Qiagen, UK), using 1 µg gDNA treated RNA as recommended by the manufacturer’s protocol. Real-time quantitative PCR (RT-qPCR) was carried out using the QuantiFast SYBR^®^ Green RT-PCR kit (Qiagen, UK) on a StepOne Plus real-time PCR system (Thermo Fisher Scientific, UK) targeting genes using primers in **Table 1**. The primer sequences for *gloverin*, *cecropin*, *hemolin*, insect metalloproteinase inhibitor (*IMPI*) and *ubiquitin* genes were acquired from prior publications (Dubovskiy et al., 2016; Lange et al., 2018b). The primer sequences for *Hdd11* were designed using NCBI primer-BLAST and their specificity were checked against the *G. mellonella* genome. Specificity of all PCR amplicons were confirmed by agarose gel electrophoresis and staining. All PCR amplicons were sequenced and blasted against the *G. mellonella* nucleotide database (NCBI), which returned expected matches [NCBI ascension number: XM_026892927 (ubiquitin), XM_026898304 (cecropin), XM_026893524 (hemolin), XM_031907851 (Hdd11), XM_026909162 (gloverin), XM_031913565 (IMPI)]. Fold changes for each gene, relative to the naïve control at t = 0 h, are shown using the comparative CT (ΔΔCt) method using *ubiquitin* as the house keeping gene. Gene expression was determined using three independent experiments, and each sample was run in triplicate. The Mann-Whitney U test was used for statistical analyses.

**Table 1:** List of primers used in this study.

Gene	Primer sequence (5′-3′)	Reference
Gloverin F	GTGTTGAGCCCGTATGGGAA	Lange et al. 2018b
Gloverin R	CCGTGCATCTGCTTGCTAAC	Lange et al., 2018b
Cecropin F	CTGTTCGTGTTCGCTTGTGT	Lange et al. 2018b
Cecropin R	GTAGCTGCTTCGCCTACCAC	Lange et al., 2018b
Hemolin F	CTCCCTCACGGAGGACAAAC	Lange et al., 2018b
Hemolin R	GCCACGCACATGTATTCACC	Lange et al., 2018b
IMPI F	TAGTAAGCAGTAGCATAGTCC	Dubovskiy et al., 2016
IMPI R	GCCATCTTCACAGTAGCA	Dubovskiy et al., 2016
Hdd11 F	TCGGCTTGTGAGTTCGTTGT	This study
Hdd11 R	GGCACTAGAAGGAGCACCAC	This study
Ubiquitin F	TCAATGCAAGTAGTCCGGTTC	Lange et al., 2018b
Ubiquitin R	CCAGTCTGCTGCTGATAAACC	Lange et al., 2018b

## Results

### Larval Survival Following Infection With BCG *lux*

**Figure 1:**
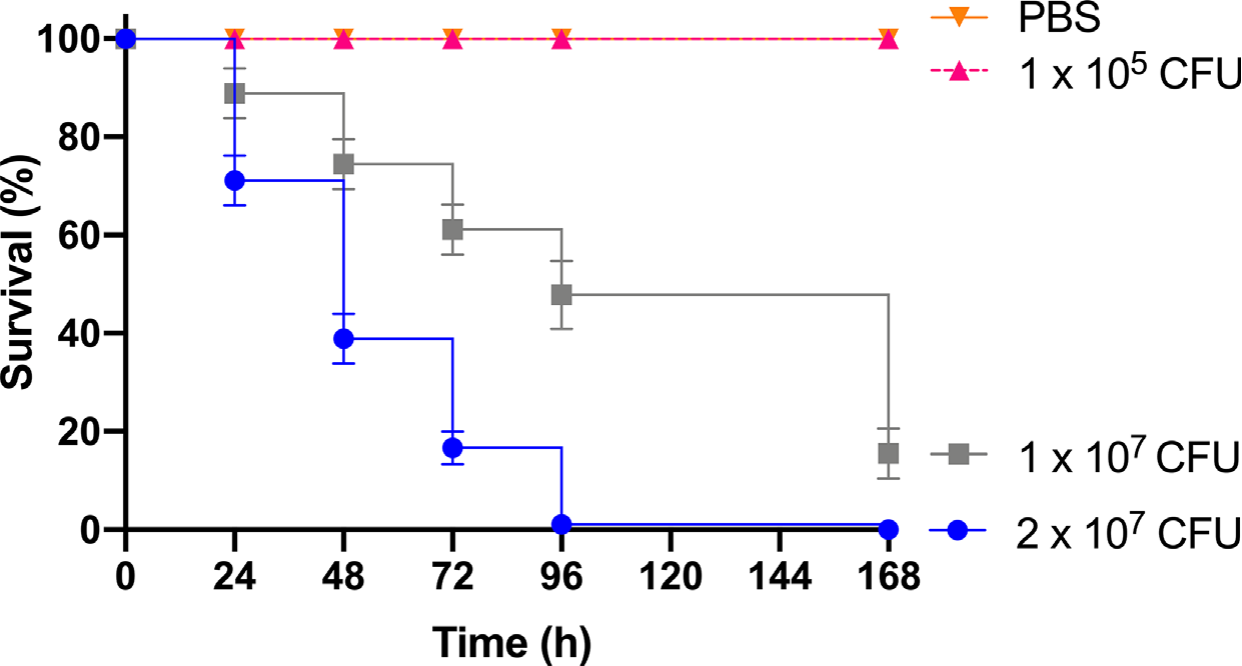
Survival curve of *G. mellonella* over a 168 h time course following infection with varying BCG *lux* inocula. Data represent three independent experiments, n=30 for each experimental group. Plotted are the mean and the standard deviation of the mean.

**Figure 2:**
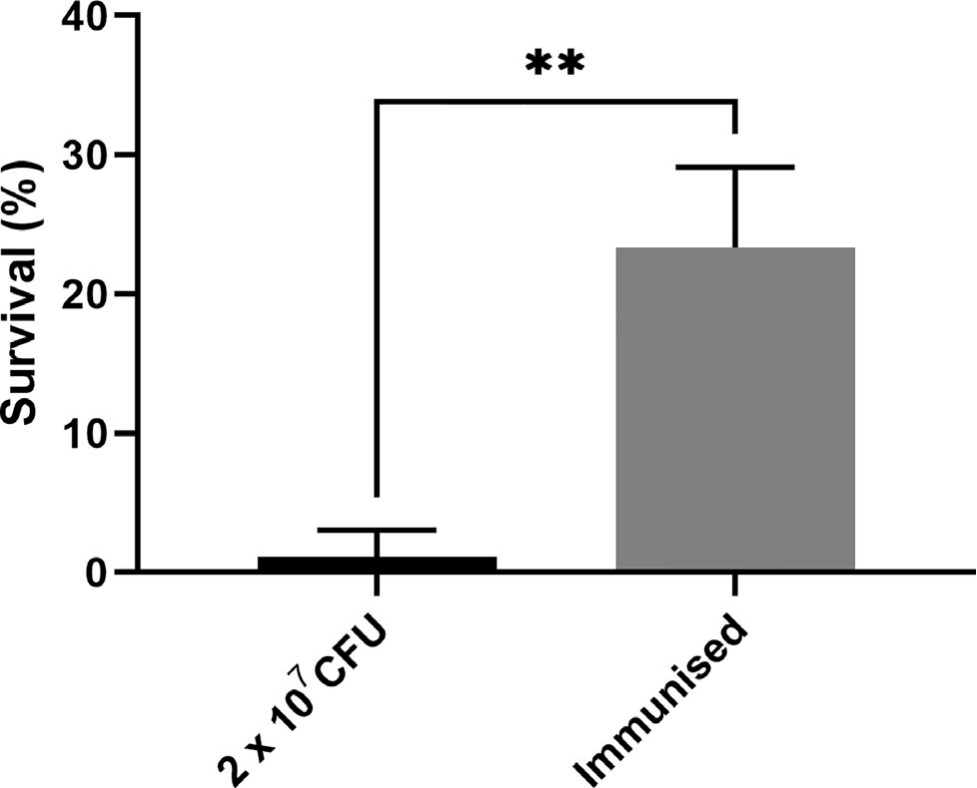
Survival of *G. mellonella* 96 h post infection immunized with a non-lethal dose of BCG *lux* 48 h pre infection. Data represent three independent experiments n = 30, n = 10 for each experimental repeat. Plotted are the mean and the standard deviation of the mean. ** = p < 0.01.

As previous work focused on larval and BCG *lux* survival over a time course of 96 h (Li et al., 2018; Asai et al., 2019a; Asai et al., 2019b), larval survival was determined over a longer 168 h time course for this proteomic and transcriptomic analysis (**Figure 1**). Larvae were considered dead when they failed to respond to touch. Larval survival decreased with increasing BCG *lux* inoculum density, where 2 x 10^7^, 1 x 10^7^, and 1 x 10^5^ CFU resulted in 0, ~50 and 100% survival at 96 h pi, respectively. By 168 h pi, larvae infected with 1 x 10^7^ and 1 x 10^5^ CFU had ~15% and 100% survival, respectively. The PBS-T group displayed no fatalities, suggesting that neither injection procedure nor PBS-T induced adverse effects on survival outcome of the larvae. However, by 168 h pi some larvae in the 1 x 10^5^ CFU and PBS-T groups began to pupate, entering their next stage of life cycle. These larvae were removed from the group and were omitted from the study. Over the course of infection larvae began to melanize at 48 h pi, which progressed steadily to extensive melanization at 96 h - 168 h pi. Larvae immunized with 1 x 10^5^ CFU BCG *lux*, 48 h prior to infection with 2 x 10^7^ CFU BCG *lux*, displayed a significant improvement in survival outcome at 96 h pi (t-test, p < 0.01) compared to those non-immunized (30% vs 3% respectively, **Figure 2**).

### Survival of BCG *lux* Within *G. mellonella*

**Figure 3:**
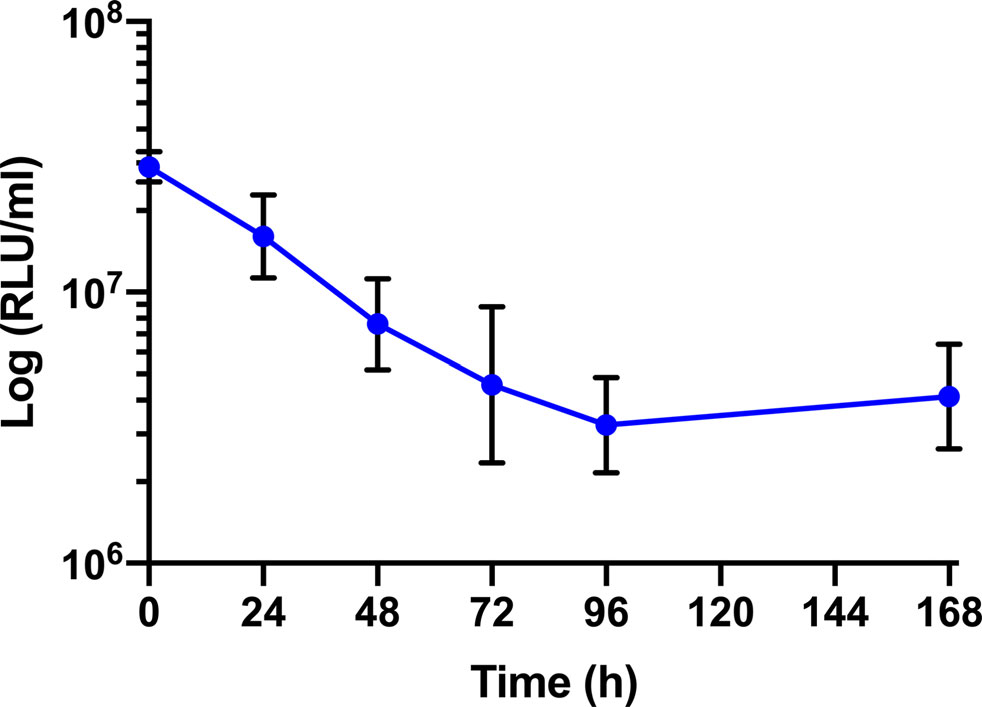
*In vivo* survival of BCG *lux* in *G. mellonella*. Changes in BCG *lux* bioluminescence (relative light units, RLU) within *G. mellonella* following infection (1 x 10^7^ CFU) over a 168 h time course. Data represent three independent experiments each with five technical replicates. Plotted are the mean and the standard deviation of the mean. RLU : CFU is approximately 4:1 as previously described by Li et al., 2018.

The survival of BCG *lux* (1 x 10^7^ CFU) within *G. mellonella* was monitored over a 168 h time course, where bioluminescence of the larval homogenate was used as a rapid and validated method of quantifying changes in mycobacterial load *in vivo* (**Figure 3**). While previous data are available (Li et al., 2018), survival was repeated to ensure consistency with the survival curve for these experiments. The bioluminescence of BCG *lux* steadily decreased over the first 72 h of infection as the bacteria likely succumbed to the initial larval innate immune response. However, between 72 and 168 h pi, the reduction in bioluminescence became more gradual and plateaued by 168 h pi. These observations, consistent with our previous data (Li et al., 2018), indicate that by 72 to 96 h pi, BCG *lux* had evaded the larval innate immune response and established a persistent infection. Survival of BCG *lux* (1 x 10^5^ CFU) was monitored similarly; at this lower dose RLU continued to decline until 96 h, where a slight increase in RLU was observed at 168 h pi (**Supplementary Figure 1**).

### Analysis of *G. mellonella* Proteome Following Infection With BCG *lux*

**Figure 4:**
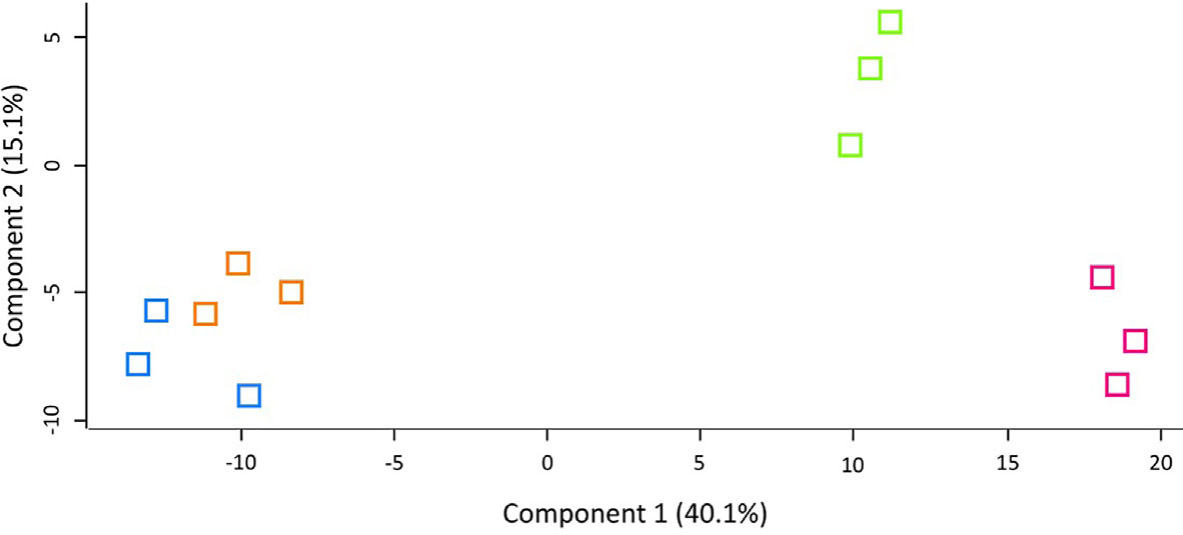
Principal component analysis (PCA) of *G. mellonella* haemolymph proteomic profiles following infection with BCG *lux* inoculum (1 x 10^7^ CFU) after 0 h (blue), 4 h (orange), 48 h (green), and 168 h (pink). Number of larvae/time point/replicate n=10. PCA of three replicates included in label free quantification analysis with a clear distinction between each time point.

LFQ proteomic analysis was carried out on larval cell free haemolymph extracted from BCG *lux* (1 x 10^7^ CFU) infected larvae at 4, 48, and 168 h pi, and the proteomic profiles were compared to that of 0 h control (naïve larvae). A total of 2013 peptides were identified representing 185 proteins with two or more peptide matches, of which 104 proteins were found at all time points. A number of differentially abundant proteins (ANOVA, p < 0.05), with a fold change of > ± 1.5 were found, i.e., 6 at 4 h pi, 54 at 48 h pi, and 86 at 168 h pi, and a total 11 proteins were deemed exclusive (LFQ signal found in all replicates of a given time point). These exclusive proteins were used in statistical analysis following imputation of the zero value as described in the methodology. Principal component analysis (PCA) of filtered proteins clearly distinguished the proteome of each time point, and clustered independent replicates of each time point together (**Figure 4**). The full list of differentially abundant proteins is available in **Supplementary Tables 1–6**.

**Figure 5:**
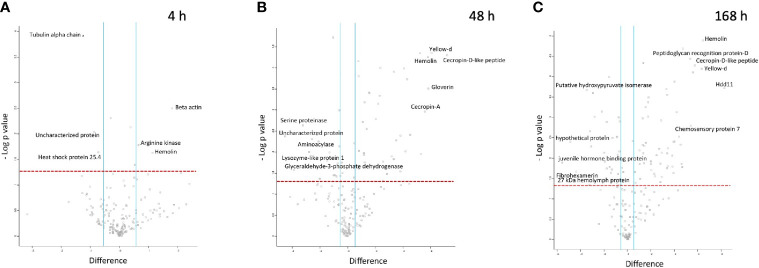
Proteomic responses of *G. mellonella* larvae following infection by BCG *lux* (1 x 10^7^ CFU) after 4 h **(A)**, 48 h **(B)**, and 168 h **(C)**. Volcano plots represent protein intensity difference (- log2 mean intensity difference) and significance in differences (- log P-value) based on a two-sided t-test. Proteins above the red dashed line are considered statistically significant (p value < 0.05) and those to the right and left of the vertical blue lines indicate relative fold changes > ± 1.5. These plots are based upon post imputed data. Each independent experiment consists of pooled haemolymph collected from 10 larvae. Data represent 3 independent experiments.

At 4 h pi with BCG *lux*, β-actin (+3.5 fold), hemolin (+2.2 fold) and arginine kinase (+1.6 fold) were found differentially more abundant in larval haemolymph compared to 0 h. Tubulin alpha chain (-2.4 fold), and heat shock protein 25.4 (-1.7 fold) were differentially less abundant in the larval haemolymph compared to 0 h (**Figure 5A**).

Larval proteins, which increased in relative abundance 48 h pi when compared to 0 h were cecropin-D-like peptide (+145.2 fold), gloverin (+56.3 fold), hemolin (+55.7 fold), cecropin-A (+47.5 fold), putative defence protein Hdd11 (+18.3 fold), serpin-4B (+14 fold), prophenoloxidase activating enzyme 3 (+14 fold), insect metalloproteinase inhibitor (+11.5 fold), serpin-11 (+5.9 fold), transgelin (+4.6 fold), Hdd-1 like protein (+2.9 fold), serpin-3a (+2.3 fold). Proteins which decreased in relative abundance 48 h pi were GAPDH (-14.7 fold), serine proteinase (-9.4 fold), lysozyme-like protein 1 (-4.74 fold), scolexin (-2.1 fold), and 27 kDa haemolymph protein (-1.9 fold) (**Figure 5B**).

Larval proteins which were increased in relative abundance at 168 h pi when compared to 0 h were putative defence protein Hdd11 (+310.1 fold), hemolin (+87.8 fold), cecropin-D-like peptide (+54.42 fold), chemosensory protein 7 (+42.2 fold), transgelin (+26.6 fold), prophenoloxidase activating enzyme 3 (+24.6 fold), serpin-4B (+20.9 fold), cecropin-A (+16.3 fold), serpin-11 (+15.1 fold), hdd1 (+10.8 fold), serpin-2 (+10.3 fold), gloverin (+7.61 fold), serpin-3a (+2.6 fold). Proteins which decreased in relative abundance 169 h pi were 27 kDa haemolymph protein (-9.6 fold), serine proteinase (-9.1 fold), GAPDH (-7.7 fold), scolexin (-1.9 fold), and apolipophorin (-1.7 fold) (**Figure 5C**).

### BCG *lux* Proteins Detected in *G. mellonella* Haemolymph

LFQ proteomics analysis additionally identified 15 BCG *lux* proteins within the haemolymph of the infected larvae during the 168 h time course. At 4 h pi, ribonuclease VapC, 2,3 bisphosphoglycerate-dependent phosphoglycerate mutase, diacylglycerol O-acyltransferase, and adenosylhomocysteinase were detected. At 48 h pi, molybdoperin, acyl-CoA dehydrogenase (FadE23), and an uncharacterised protein containing an undecaprenyl diphosphate synthase domain were detected. However, no proteins with a known function associated with virulence, intracellular metabolism or survival, were detected at 168 h pi. A list of detected BCG *lux* proteins is available in **Supplementary Table 7**. The relative abundance of these proteins could not be determined, and these data are qualitative observations.

### *G. mellonella* Gene Expression Following BCG *lux* Infection

**Figure 6:**
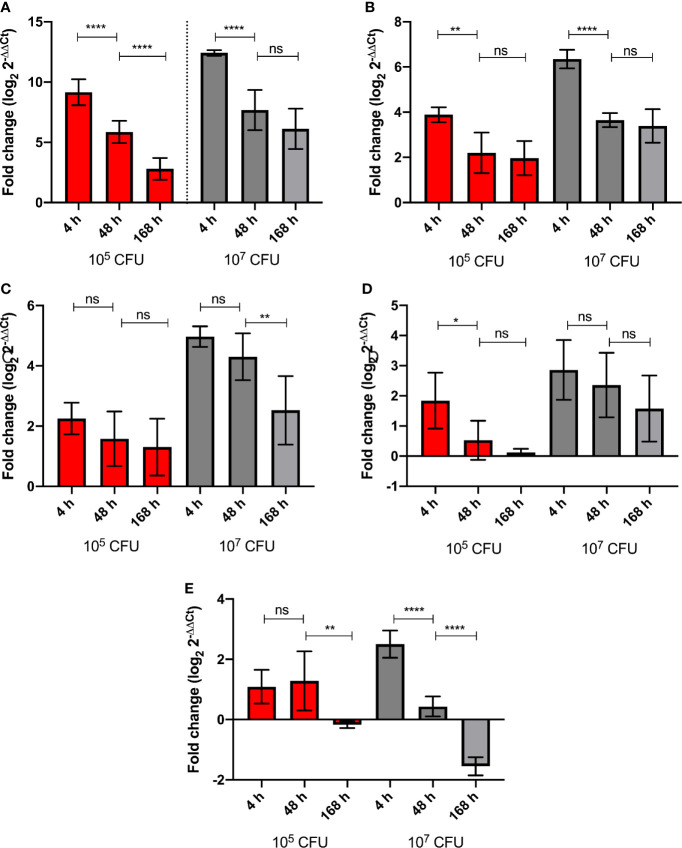
Level of *G. mellonella* gene expression was measured by RT-qPCR from RNA of larvae challenged with either low dose (10^5^ CFU, red) or high dose (10^7^ CFU, grey) BCG *lux* at 4, 48, and 168 h pi. The comparative ΔΔCt method was used for relative quantification of **(A)** *gloverin*, **(B)** *cecropin*, **(C)** *hemolin*, **(D)** *Hdd11*, and **(E)** *IMPI* using *ubiquitin* as a housekeeping gene. Data represent three independent experiments, each with RNA extracted from five larvae, which was pooled. Each independent experiment consisted of three technical repeats. Bar charts represent changes in gene expression expressed as fold change. Comparison of gene expression with each infectious dose over time was carried out using the Mann-Whitney U test; where *,**,****, and ns signifies p < 0.05, 0.01, 0.0001 and non-significant, respectively.

*G. mellonella* larvae were challenged with high (1 x 10^7^ CFU) and low (1x10^5^ CFU) doses of BCG *lux*. Over a 168 h time course, the expression of five genes of the *G. mellonella* innate immune system were measured at 4, 48, and 168 h pi, *via* RT-qPCR using the ΔΔCt method, *ubiquitin* as the housekeeping gene, and naïve non-infected larvae as the reference. Expression of the house keeping gene *ubiquitin* and four others encoding proteins that were differentially expressed in the proteomic analysis, i.e., *hemolin*, *IMPI*, *gloverin*, and *cecropin*, were measured. *Hemolin*, and its encoded protein, was found to be differentially abundant at all time points, *IMPI*, and its encoded protein, was found in relatively consistent abundance at 48 and 168 h pi, as was *Hdd11* and its encoded protein, which is of interest as it is involved in the formation of granuloma-like structures, which are a hallmark of TB infection (Sheehan et al., 2020b). For all five genes tested, the high dose BCG *lux* infection induced greater gene expression/suppression at all time points when compared to the low dose (**Supplementary Figure 2**). While none were statistically significant, all genes displayed a trend of reduction of gene expression over time. *Gloverin* and *cecropin* encode AMPs induced in *G. mellonella* in response to a number of bacterial and fungal pathogens (Tsai et al., 2016). The gene expression for the AMPs, *gloverin*, and *cecropin*, in response to high dose infection, significantly decreased between 4 h and 48 h pi (p < 0.0001). However, the level of gene expression was lower but of similar levels between 48 and 168 h pi for both genes (**Figures 6A, B**). For low dose expression *gloverin*, expression continued to decrease significantly between 4, 48, and 168 h pi (p < 0.0001), while *cecropin* mirrored high dose infection but at significantly lower levels of gene expression (**Figures 6A, B**, **Supplementary Figures 2A, B**). For the *hemolin* gene, high dose infection induced statistically significant reduction in gene expression between 48 and 168 h pi (**Figure 6C**) (p < 0.01). No significant reduction in gene expression was observed in low dose infection. High dose infection resulted in statistically significant higher levels of gene expression at all time points when compared to low dose infection (**Supplementary Figure 2C**) (P < 0.001 for 4 and 48 h pi and P < 0.05 for 168 h pi). For *Hdd11*, high dose infection induced greater levels of gene expression with a trend of reduction for all time points (**Figure 6D** and **Supplementary Figure 2D**). Finally, for the *IMPI* gene, induction of gene expression followed high dose infection and thereafter steadily decreased over time (**Figure 6E**), with apparent suppression between 48 and 168 h pi (p < 0.0001). Low dose infection led to a sustained level of gene induction between 4 and 48 h pi, and a reduced substantial gene suppression between 48 and 168 h pi (p < 0.01).

## Discussion

Previously we described, using survival curves, bacterial counts, and electron microscopy, a BCG *lux* - *G. mellonella* infection model for the MTBC complex (Li et al., 2018; Asai et al., 2019a; Asai et al., 2019b). The aim of this study was to increase our understanding of the host-bacterial interactive biology, focusing on innate immunity, through proteomic and gene expression analyses. To accommodate the increased incubation period in this study, larval survival and changes in BCG *lux* load *in vivo* over the 168 h time course were examined, which was consistent with expected observations extrapolated from our prior studies (Li et al., 2018; Asai et al., 2019a; Asai et al., 2019b). Using this established model of BCG persistence, we characterised the larval innate immune response to BCG *lux* (1 x 10^7^ CFU) at 4 h, 48 h, and 168 h pi using LFQ proteomics. Distinct changes in larval proteome in response to infection at all three time points were found, with the least changes in differentially abundant proteins being at 4 h pi.

Hemolin, a member of the immunoglobulin super family (Eleftherianos et al., 2007), was found to be more abundant in the infected larvae at all time points with increasing abundance over time, 4 h (+2.2 fold) 48 h (+55.7 fold) and 168 h (+87.8 fold) pi. Hemolin aids haemocyte aggregation and phagocytosis of foreign cells through recognition of lipopolysaccharide (LPS) and lipoteichoic acid (LTA) of Gram-negative and -positive bacteria, as well as fungal β-1,3-glucan (Eleftherianos et al., 2007; Jung et al., 2019). Hemolin opsonizes microbial pathogens and binds to haemocytes, enhancing phagocytic uptake and eliminating invading pathogens (Aathmanathan et al., 2018). The interaction of hemolin with the structurally unique mycobacterial cell wall has not been described. However, as hemolin is able to recognize microbial molecular signatures, we speculate that *G. mellonella* hemolin may detect lipoglycans (noncanonical type V LTA) found in abundance on the mycobacterial cell wall (Mishra et al., 2011); this will be investigated in a future study.

As an intracellular pathogen, exploiting innate immune mechanisms to increase phagocytic uptake would be beneficial to mycobacteria. The increased abundance of β-actin at 4 h pi (+3.5 fold), may reflect the cytoskeletal redistribution and remodelling which takes place during phagocytosis (Rougerie et al., 2013). In line with this observation, infection of primary murine epithelial and lung cells with BCG has been shown to up-regulate actin redistribution (Alaridah et al., 2017). Studies of early granuloma structures in larval zebrafish found extensive reorganization of the actin cytoskeleton in granulomatous macrophages (Cronan et al., 2016). Therefore, the abundance of β-actin at 48 h and 168 h pi (+5.0 and +18.6 fold) may reflect haemocyte actin reorganization within developing granuloma-like structures. The presence and increasing abundance of β-actin in the cell-free haemolymph over time, likely reflects the equilibrium between live and dead host-cells in the granuloma-like structure (Cronan et al., 2018), with necrotic and/or lytic haemocytes likely releasing β-actin into the haemolymph.

AMPs were induced following infection with BCG *lux*. Cecropin A, cecropin-D-like protein, and gloverin were differentially more abundant than at 0 h at both 48 h and 168 h pi, but not at 4 h pi. Cecropins are amphipathic α-helical AMPs, reported to be effective against both Gram-positive and Gram-negative bacteria by permeating the bacterial cell wall (Zdybicka-Barabas et al., 2019). Gloverin is a glycine rich AMP efficacious against filamentous fungi and Gram-positive and Gram-negative bacteria (Wojda, 2017). As a relative comparison, gloverin (+121.7 fold), cecropin-D-like peptide (+73.7 fold), and cecropin-A (+10.56 fold) were also more abundant in larvae infected with 2 x 10^6^ CFU of *S. aureus* 24 h pi (Sheehan et al., 2019). BCG *lux* infection led to increased expression of cecropin-D-like peptide (+145.2 fold), gloverin (+56.3 fold), and cecropin-A (+47.5 fold) at 48 h pi. Although variance in the infectious dose and time point complicates comparisons, the higher abundance of gloverin in response to a lower infectious dose of *S. aureus* may indicate that gloverin is preferentially expressed in the presence of Gram-positive bacteria, or alternatively the higher abundance of cecropin-D-like peptide may indicate BCG *lux* is a more potent inducer. In mammals, cathelicidins, cationic α-helical AMPs, are released by leukocytes in response to infection (Arranz-Trullén et al., 2017). The human cationic antimicrobial peptide 18 (h-CAP-18) is the leading AMP in TB therapeutics, with both bactericidal and immunomodulating activity (Arranz-Trullén et al., 2017). Immunomodulation of macrophages and neutrophils by LL-37, a proteolyzed component of h-CAP-18, has been reported during infection, such as the promotion of autophagy in human monocytes (Arranz-Trullén et al., 2017). A previous report indicates that Cecropin-A induces immunomodulating activity in macrophages, regulating inflammatory responses (Lee et al., 2015a). If similar activities occur in phagocytic haemocytes, then increased abundance of hemolin and cytoskeletal actin could result from upregulation of autophagy-related activities. The production of synthetic cecropin-D-like peptides has been reported (Correa et al., 2014b; Correa et al., 2014a). Cytotoxicity of cecropin-D-like peptides, has previously been determined against erythrocytes with haemolysis of 2.3% at 115 µM (Oñate-Garzón et al., 2017). Cecropin-D-like peptides have shown antimicrobial activity against Gram-positive (*S. aureus*) and negative (*Pseudomonas aeruginosa* and *E. coli*) bacteria through permeabilization and disruption of the cell membrane (Cytryńska et al., 2007; Oñate-Garzón et al., 2017). Therefore, *in vitro* drug screening against mycobacteria using synthetic *G. mellonella* AMPs, may reveal promising antimycobacterial properties.

Hdd11 (homologous to Noduler) is associated with the formation of nodules or granuloma-like structures; facilitated by crosslinking pathogens and haemocytes by Hdd11 (Gandhe et al., 2007; Sheehan et al., 2020b). In line with published histopathology data from BCG *lux* infected *G. mellonella* larvae (Li et al., 2018), where numbers of granuloma-like structures increased over time, the abundance of Hdd11 within the haemolymph increased +18.3 and +310 fold compared to 0 h, as infection progressed at 48 h and 169 h pi, respectively. The fungal granulomatous disease, mycetoma, caused by *M. mycetomatis* has been modelled using *G. mellonella* larvae. In larvae infected with *M. mycetomatis*, Hdd11 was +533 fold more abundant within the haemolymph at 168 h pi when compared to 0 h (Sheehan et al., 2020b). Although Hdd11 levels are higher in *M. mycetomatis* infection, this indicates that BCG *lux* induces a similar granulomatous response. Formation of early granuloma in humans is tightly regulated by the interaction between matrix metalloproteinases (MMPs) and tissue inhibitors of MMPs (TIMPs) (Parasa et al., 2017). IMPI, found differentially abundant at 48 h and 168 h pi, is an insect metalloproteinase inhibitor, but has no structural similarities to vertebrate or invertebrate TIMPs (Vilcinskas and Wedde, 2002). IMPI does not inhibit host MMP activity, its primary function is to inhibit microbial metalloproteinases. However, IMPI can additionally regulate endogenous metalloproteinases involved in metamorphosis and thereby, depending on the life stage of the larvae, potentially impact on the larval tissue response to infection (Wedde et al., 2007). MMPs and TIMPs have been identified in fruit flies, and it is likely similar systems exist in *G. mellonella* (Vilcinskas and Wedde, 2002). The challenge remains in identifying these systems, as the publicly available genome of *G. mellonella* is yet to be fully annotated (Lange et al., 2018a).

Another key invertebrate innate immune response is melanization, modulated through the PO cascade (Dubovskiy et al., 2013). Proteins associated with the PO cascade were more abundant at 48 h pi (phenoloxidase activating enzyme 3, serpin-3a, serpin-4b, and serpin-11) and 168 h pi (phenoloxidase activating enzyme 3, serpin-2, serpin-3a, serpin-4b, and serpin-11), but not at 4 h pi. In contrast, larvae challenged with a lower infectious dose of *S. aureus* (2 x 10^6^ CFU) induced more abundant levels of phenoloxidase activating enzyme 3 and serpin-4b by 6 h pi compared to the 0 h control (Sheehan et al., 2019). This may reflect the more virulent or rapid growth of *S. aureus*, as the rate of mortality was 90% by 72 h pi (Sheehan et al., 2019). Previous studies using the *G. mellonella* – BCG *lux* infection model, found slow gradual melanization over 96 h, with little to no melanization within the first 24 h (Asai et al., 2019a). Serpins, a class of protease inhibitor which negatively regulates the PO cascade (Lu et al., 2014), were detected in response to BCG *lux* infection. We speculate that the lack of abundant PO cascade proteins at 4 h pi and the rate of larval melanization, reflects the slow growth and persistence of BCG *lux* infection leading to host modulation of the PO cascade to prevent uncontrollable melanization.

While distinct changes in proteomic signatures over the time course were detected, changes specific to mycobacteria infection were not identified. Despite the lack of a BCG specific response, *G. mellonella* larvae immunized with a non-lethal dose of BCG *lux* (1x10^5^ CFU), 48 h prior to infection with a lethal dose of BCG *lux* (2x10^7^ CFU), resulted in improved survival outcome when compared to the non-immunized control. This observation, commonly referred to as immune priming, has widely been reported in invertebrate infection models (Bergin et al., 2006; Sadd and Schmid-Hempel, 2006; Pham et al., 2007; Sheehan et al., 2020a). The precise mechanism, of how a non-lethal exposure to a pathogen leads to protection/immunity, remains unclear with a number of mechanisms being suggested (Sheehan et al., 2020a). However, it is known that non-lethal exposure typically leads to an increase in the number of circulating haemocytes and the production of AMPs (Bergin et al., 2006). Moreover, it is unclear as to whether these responses are pathogen type/class specific or are more generalized. Therefore, future studies will focus on whether immunization with non-lethal BCG dose leads to primed protection against other mycobacterial species, Gram-positive/negative bacteria and fungi.

LFQ proteomics analysis identified BCG *lux* proteins within the larval haemolymph giving qualitative insight into the response of the larval innate immune system. Most proteins detected were associated with intracellular adaptations and survival. At 4 h pi, proteins were associated with metabolic changes. These included: ribonuclease VapC and 2,3 bisphosphoglycerate-dependent phosphoglycerate mutase, both of which have been associated with regulation of metabolism in response to intracellular conditions and glucogenesis (Hillion et al., 2017; Sharrock et al., 2018). Adenosylhomocysteinase catalyses the reversible processing of S-adenosylmethionine dependent methyltransferase reaction and is essential for *in vitro* growth, and was upregulated in lung tissues of MTB infected mice (Singhal et al., 2012). Diacylglycerol O-acyltransferase is involved in the accumulation of triacylglycerol in MTB under stress (Sirakova et al., 2006). The presence of diacylglycerol O-acyltransferase is in agreement with the accumulation of lipid droplet structures in BCG *lux in vivo* in *G. mellonella* which have been observed using transmission electron microscopy (Li et al., 2018). At 48 h pi, proteins associated with intracellular survival were detected. Molybdoperin is induced in response to hypoxia and nitric oxide stress (Wang et al., 2013). Acyl-CoA dehydrogenase induced in response to cell envelope damage following oxidative and nitrosative stress, may have a role in recycling of fatty acids (Voskuil, 2013). An uncharacterised protein with an undecaprenyl diphosphate synthase domain, typically associated with cell wall synthesis was detected (Kaur et al., 2004). Abundance of this protein may be associated with thickening of the mycobacterial cell wall as a stress response to intracellular conditions (Ghazaei, 2018). Similar to 4 h pi, diacylglycerol O-acyltransferase was also detected at 48 h pi. These proteins in the haemolymph may be associated with MTB extracellular vesicles, as both diacylglycerol O-acyltransferase and acyl-CoA dehydrogenase have been detected in such structures. (Lee et al., 2015b). The presence of BCG *lux* proteins involved in intracellular adaptation and survival at 4 and 48 h pi indicate that the haemocytic environment is a mycobacterial stress-inducer.

The *G. mellonella* innate immune response to BCG *lux* was also investigated using RT-qPCR. The results show that infection with BCG *lux* at high (1 x 10^7^ CFU) and low (1x10^5^ CFU) doses, led to differential gene expression of innate immune genes, with higher dose infection inducing more substantial changes. Changes in gene expression were detected in *gloverin*, *cecropin*, *IMPI*, *hemolin*, and *Hdd11* regardless of infectious dose when compared to the 0 h control. Induction of expression was highest at 4 h pi, and thereafter decreased over the time course despite the presence of a persistent infection. Similar observations were made in an RT-qPCR study of *G. mellonella* innate immune responses to infection with *Riemerella anatipestifer*, a Gram-negative bacterium, where genes involved in innate immunity (e.g., encoding AMPs and opsonin), were highest at 4 h pi, and declined over time (Liu et al., 2019). In this study, the trend between the presence of protein and the corresponding mRNA improved over the course of infection, but the abundance of protein was largely inconsistent with gene expression signals, with only 20% in agreement between 48-168 h pi. Proteins induced by genes expression at 4 h pi were mostly undetected in the proteomic analysis with the exception to hemolin. The discrepancy between the two datasets may be attributed to the preparation of the samples. The proteomic study was carried out on cell free haemolymph, whereas the gene expression study was carried out on mRNA prepared from the whole larva. Gene expression studies in both humans (Sonawane et al., 2017) and insects, *Manduca sexta* (Cao and Jiang, 2017) and *Ceracris nigricornis* Walker (Yuan et al., 2019), have highlighted the importance of acknowledging tissue specific expression of genes. It is unknown whether the genes examined in this study are expressed globally or are tissue specific. Nevertheless, the comparison of proteomics and transcriptomics has been an ongoing subject of discussion. A study that compared the correlation of proteome and transcriptome across developmental stages of fruit flies (Becker et al., 2018) and *Epicauta chinensis* (blister beetle) (Li et al., 2014) reported a low correlation (20%–34%) between mRNA levels and protein expression. Becker et al. 2018 adjusted for the translational delay of mRNA into protein (~ 4-6 h) for a small subset of genes in fruit flies, which showed modest improvements (+2.4%). This supports the idea that there are lags between the initiation of gene expression and protein biosynthesis, possibly due to the basal mRNA state (steady, long/short-term), downstream gene regulation, post-transcriptional modification to mRNA, and/or availability of biosynthetic material (Liu et al., 2016). Studies have determined that the correlation between these two Omics approaches, are modest at best (40%) (Gygi et al., 1999; Kumar et al., 2016), with correlation decreasing with increasing biological complexity, i.e., single cellular to multi-cellular organisms (Gygi et al., 1999; De Sousa Abreu et al., 2009; Schwanhüusser et al., 2011; Kumar et al., 2016; Meir et al., 2018). Furthermore, a comparative proteomic and transcriptomic study of 29 human tissue types revealed that a number of protein molecules synthesised per highly abundant transcript were near quadratic (Wang et al., 2019). It is generally speculated that abundance of protein/gene is regulated at the translational level through enhanced translational efficiency and protein stability (Vogel et al., 2010). The presentation of both the gene expression and proteomic data in this paper is the first of its kind for *G. mellonella*. We acknowledge that the number of genes tested are limited and a more comprehensive analysis to determine a broader correlation between transcriptome and proteome of *G. mellonella* is warranted in the future. As proteomics investigates the presence/absence and relative abundance of a protein, it is a more reliable indicator of the state of the innate immune response of *G. mellonella* at a given time point.

In conclusion, to our knowledge, this is the first report of the innate immune response of *G. mellonella* to mycobacterial infection using molecular transcriptomic and proteomic approaches. The proteomic profiles were unique over the course of infection. Responses were similar to those in *G. mellonella* infected with fungal granulomatous infectious disease. BCG *lux* proteins were detected, associated with intracellular survival and metabolism in vertebrate hosts, indicating that *G. mellonella* induces similar stress responses. RT-qPCR confirmed the proteomic analysis. However, gene expression and protein abundance was not always in agreement, and careful comparison of data should be made in future studies. Several targets for further studies, such hemolin as an opsonising agent and cecropin as an antimycobacterial agent, were identified and will be further investigated. While this study focused on the host response to infection, future studies will evaluate the mycobacterial response to humoral innate immunity of *G. mellonella*. The data presented here provide a vital insight into the *G. mellonella* – mycobacterial infection model and highlights its value for accelerating in TB research.

## Data Availability Statement

The datasets presented in this study can be found in online repositories. The names of the repository/repositories and accession number(s) can be found in the article/**Supplementary Material**.

## Author Contributions

MA, GS, YL, BR, KK, PL, and SN conceptualized the study. MA drafted the manuscript. MA, GS, and YL carried out the experimental procedures. MA and GS carried out experimental analysis. MA, GS, YL, BR, KK, PR, and SN reviewed, edited, and approved the manuscript for submission. All authors contributed to the article and approved the submitted version.

## Funding

This work was supported by grants from the Biotechnology and Biological Sciences Research Centre (BBSRC) [BB/P001262/1] to PL and YL, and the National Centre for the Replacement, Refinement and Reduction of Animals in Research (NC3Rs) [NC/R001596/1] to PL, SN, BR, and YL. GS is the recipient of a Maynooth University doctoral studentship. The Q-exactive mass spectrometer was funded under the Science Foundation Ireland (SFI) Research Infrastructure Call 2012, [12/RI/2346] (3). This publication emanated from research supported in part by a research grant from the SFI and is co-funded under the European Regional Development Fund [12/RC/2275_P2].

## Conflict of Interest

The authors declare that the research was conducted in the absence of any commercial or financial relationships that could be construed as a potential conflict of interest.

## Abbreviations

AMP, Antimicrobial peptides; BCG, Bacillus Calmette-Guérin; IMPI, Insect metalloproteinase inhibitor; LFQ, Label free quantification; MTB, *Mycobacterium tuberculosis*; MTBC, *Mycobacterium tuberculosis* complex; NTM, Non-tuberculous mycobacterium; PAMP/DAMP, Pathogen/damage associated molecular patterns; PPR, Pattern recognition receptors; PO, Phenoloxidase; ROS, Reactive oxygen species; RT-qPCR, Reverse transcriptase quantitative PCR; TB, Tuberculosis.
