# Supplementary figures and images for: Innate Immune Responses of Galleria mellonella to Mycobacterium bovis BCG Challenge Identified Using Proteomic and Molecular Approaches
Source: Front Cell Infect Microbiol. 2021 Feb 9;11:619981. doi: 10.3389/fcimb.2021.619981 (PMC7900627; doi:10.3389/fcimb.2021.619981)

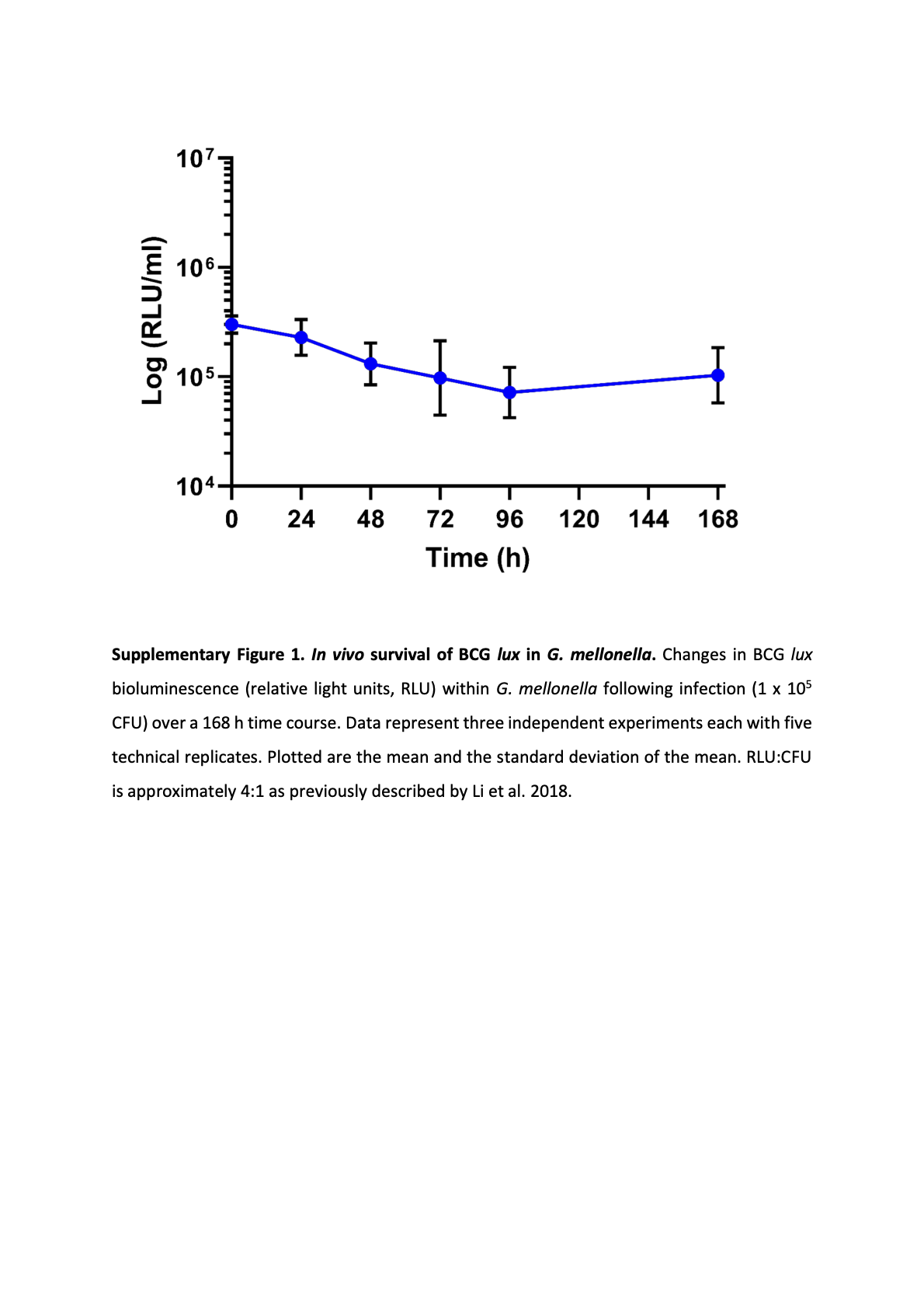

Supplement: Supplementary file 1 [file Image_1.tiff]

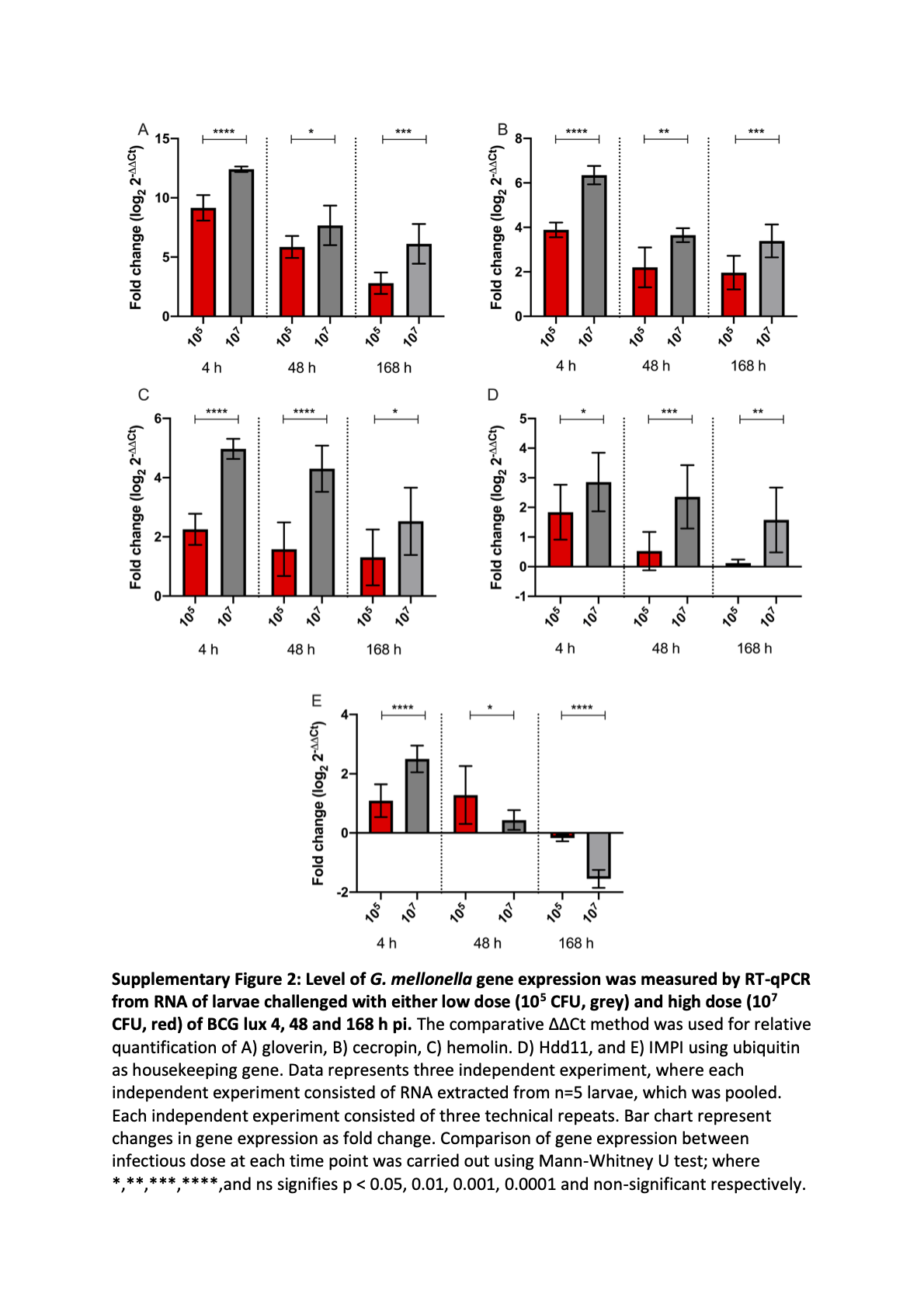

Supplement: Supplementary file 2 [file Image_2.tiff]
