# Supplementary material for: Innate Immune Responses of Galleria mellonella to Mycobacterium bovis BCG Challenge Identified Using Proteomic and Molecular Approaches
Source: Front Cell Infect Microbiol. 2021 Feb 9;11:619981. doi: 10.3389/fcimb.2021.619981 (PMC7900627; doi:10.3389/fcimb.2021.619981)
Supplement: Supplementary file 3 [file Table_1.docx]

***G. mellonella*-BCG *lux* infection model proteomics**

**Supplementary Table 1: Proteins which are increased in abundance in *Galleria mellonella* larvae hemolymph infected with BCG *lux* 1x10^7^ CFU (10 μl inoculum) at 4 h as compared to 0 h control uninfected larvae.**

| p value | Fold change (+) | GI number | Protein name | Peptides | Sequence coverage [%] | Score |
| --- | --- | --- | --- | --- | --- | --- |
| 0.003166 | 3.49 | 157927723 | Beta actin | 11 | 54.1 | 277.07 |
| 0.023588 | 2.20 | 186703381 | Hemolin | 7 | 50.7 | 323.31 |
| 0.016673 | 1.58 | 219552448 | Arginine kinase | 8 | 62.5 | 201.49 |

**Supplementary Table 2: Proteins which are decreased in abundance in *G. mellonella* larvae hemolymph infected with BCG *lux* 1x10^7^ CFU (10 μl inoculum) at 4 h as compared to 0 h control uninfected larvae.**

| p value | Fold change (-) | GI number | Protein name | Peptides | Sequence coverage [%] | Score |
| --- | --- | --- | --- | --- | --- | --- |
| 0.000121 | 2.37 | 225031010 | Tubulin alpha chain | 3 | 46.2 | 26.541 |
| 0.009208 | 1.82 | 229002332 | Uncharacterized protein | 3 | 14.5 | 114.37 |
| 0.02304 | 1.65 | 169646838 | Heat shock protein 25.4 | 13 | 48 | 237.16 |

**Supplementary Table 3: Proteins which are increased in abundance in *G. mellonella* larvae hemolymph infected with BCG *lux* 1x10^7^ CFU (10 μl inoculum) at 48 h as compared to 0 h control uninfected larvae.**

| p value | Fold change (+) | GI Number | Protein names | Peptides | Sequence coverage [%] | Score |
| --- | --- | --- | --- | --- | --- | --- |
| 4.97E-05 | 145.25 | 156630481 | Cecropin-D-like peptide | 2 | 14.5 | 46.071 |
| 4.46E-05 | 66.41 | 112984054 | Yellow-d | 18 | 44.7 | 323.31 |
| 0.000311 | 56.27 | 110649240 | Gloverin | 2 | 28.8 | 18.817 |
| 5.49E-05 | 55.73 | 186703381 | Hemolin | 7 | 50.7 | 323.31 |
| 0.001092 | 47.51 | 116087 | Cecropin-A (Cecropin-C) | 1 | 8.5 | 9.783 |
| 0.011076 | 39.12 | 23208535 | Peptidoglycan recognition-like protein B (fragment) | 13 | 34 | 231.94 |
| 4.51E-05 | 37.41 | 55139149 | Hemolin (fragment) | 18 | 58 | 323.31 |
| 0.003829 | 22.85 | 110347843 | Protease inhibitor-like protein | 2 | 40 | 31.579 |
| 0.000188 | 19.60 | 112983052 | Chemosensory protein 7; Chemosensory protein11 | 7 | 27.7 | 169.29 |
| 0.009535 | 18.28 | 74873244 | Putative defense protein Hdd11 (*Hyphantria* differentially displayed gene 11) | 9 | 60.7 | 323.31 |
| 0.000104 | 16.78 | 154240658 | Peptidoglycan recognition protein-D | 7 | 22 | 120.85 |
| 0.004092 | 14.05 | 45594226 | Serpin-4B | 4 | 70 | 68.718 |
| 0.028322 | 14.04 | 56718390 | Prophenol oxidase activating enzyme 3 | 17 | 42.1 | 210.04 |
| 0.005167 | 11.47 | 33860163 | Inducible metalloproteinase inhibitor protein [cleaved into: IMPI alpha] | 8 | 33.2 | 67.368 |
| 0.000123 | 10.10 | 112983994 | Peptidoglycan recognition protein | 13 | 57.9 | 306.89 |
| 0.014612 | 9.93 | 25989211 | CLIP domain-containing serine protease 2 | 7 | 53.6 | 102.38 |
| 0.01252 | 9.06 | 112702923 | Peptidoglycan-recognition protein-LB (fragment) | 2 | 60.5 | 41.555 |
| 0.022459 | 8.72 | 23208526 | 6tox | 6 | 17 | 36.001 |
| 0.032869 | 7.07 | 1705742 | Hyphancin-3E (Cecropin-A1) (Hyphancin-IIIE) | 1 | 4.7 | 18.42 |
| 0.047493 | 5.88 | 22901764 | Kunitz-like protease inhibitor | 3 | 23.8 | 62.084 |
| 0.016888 | 5.86 | 226342884 | Serpin-11 | 13 | 39.2 | 166.87 |
| 1E-04 | 5.16 | 125629089 | Gustatory receptor | 2 | 14.9 | 24.41 |
| 0.000461 | 4.98 | 157927723 | Beta actin | 11 | 54.1 | 277.07 |
| 0.000708 | 4.59 | 183979237 | Transgelin | 6 | 21.1 | 169.94 |
| 0.001639 | 4.08 | 52782700 | Beta-1,3-glucan-binding protein (BGBP) (Beta-1,3-glucan recognition protein) (Beta-GRP) (Gram negative bacteria-binding protein) (fragment) | 2 | 12.7 | 43.006 |
| 0.0004 | 2.95 | 157704337 | Hdd1-like protein | 6 | 56.9 | 146.94 |
| 0.002815 | 2.58 | 52782700 | Beta-1,3-glucan-binding protein (BGBP) (Beta-1,3-glucan recognition protein) (BetaGRP) (Gram negative bacteria-binding protein) (fragment) | 2 | 14.5 | 39.877 |
| 0.017544 | 2.33 | 110826028 | Esterase | 3 | 8.1 | 32.225 |
| 0.000655 | 2.32 | 156968291 | Uncharacterized protein (fragment) | 16 | 35.2 | 244.53 |
| 0.011504 | 2.28 | 158289805 | AGAP010733-PA (fragment) | 12 | 38.1 | 130.56 |
| 0.010426 | 2.26 | 27733415 | Serpin 3a (Serpin 3b) | 11 | 12.8 | 243.76 |
| 0.00195 | 2.15 | 73671225 | Superoxide dismutase [Cu-Zn] (EC 1.15.1.1) | 3 | 57.8 | 312.25 |
| 0.009242 | 1.76 | 114649525 | Beta-1,3-glucan recognition protein | 14 | 37 | 163.22 |
| 0.024575 | 1.76 | 220902980 | Carboxylic ester hydrolase (EC 3.1.1.-) | 6 | 36.2 | 158.78 |
| 0.000161 | 1.74 | 170036627 | Gelsolin (Gelsolin precursor) | 22 | 35.8 | 323.31 |
| 6.84E-05 | 1.72 | 189181680 | Carboxylic ester hydrolase (EC 3.1.1.-) | 19 | 49.1 | 323.31 |
| 0.021036 | 1.52 | 242004672 | Uncharacterized protein | 10 | 51.9 | 205.8 |

**Supplementary Table 4: Proteins which are decreased in abundance in *G. mellonella* larvae hemolymph infected with BCG *lux* 1x10^7^ CFU (10 μl inoculum) at 48 h as compared to 0 h control uninfected larvae.**

| p value | Fold change (-) | GI Number | Protein names | Peptides | Sequence coverage [%] | Score |
| --- | --- | --- | --- | --- | --- | --- |
| 0.004211 | 22.90 | 229002332 | Uncharacterized protein (fragment) | 3 | 14.5 | 114.37 |
| 0.0273 | 14.68 | 109119903 | Glyceraldehyde-3-phosphate dehydrogenase (EC 1.2.1.12) | 13 | 51.2 | 192.01 |
| 0.002301 | 9.39 | 121308868 | Trypsin-like serine proteinase | 9 | 25.4 | 96.748 |
| 0.009875 | 6.84 | 112983816 | Glyceraldehyde-3-phosphate dehydrogenase (EC 1.2.1.12) | 8 | 38.8 | 67.248 |
| 0.004925 | 5.96 | 116782397 | Uncharacterized protein | 8 | 16.9 | 84.718 |
| 0.023861 | 4.74 | 145286562 | Lysozyme-like protein 1 | 3 | 9.2 | 48.058 |
| 0.003929 | 4.69 | 77415676 | Uncharacterized protein | 15 | 32.2 | 323.31 |
| 0.005601 | 4.30 | 114052174 | Aminoacylase | 6 | 20.1 | 38.514 |
| 0.016362 | 2.78 | 116791778 | Uncharacterized protein | 22 | 51.8 | 323.31 |
| 0.010089 | 2.48 | 164459610 | Putative hydroxypyruvate isomerase (EC 5.3.1.22) | 13 | 33.3 | 109.06 |
| 1.9E-05 | 2.07 | 153791206 | Scolexin | 15 | 28.2 | 323.31 |
| 0.035455 | 2.03 | 183979239 | Similar to CG10638-PA | 4 | 28.8 | 145.1 |
| 0.03539 | 1.87 | 46396271 | 27 kDa hemolymph protein (P27K) (27k) | 13 | 59.6 | 323.31 |
| 0.037852 | 1.74 | 114052174 | Aminoacylase | 6 | 17.8 | 52.484 |
| 0.018555 | 1.66 | 134103857 | Cationic peptide CP8 | 6 | 29.2 | 323.31 |
| 0.001004 | 1.53 | 112983062 | Lipopolysaccharide binding protein | 13 | 47.7 | 173.12 |
| 0.007223 | 1.52 | 158515746 | Juvenile hormone binding protein | 15 | 41.7 | 323.31 |

**Supplementary Table 5: Proteins which are increased in abundance in *G. mellonella* larvae hemolymph infected with BCG *lux* 1x10^7^ CFU (10 μl inoculum) at 168 h as compared to 0 h control uninfected larvae.**

| p value | Fold Change (+) | GI Number | Protein Name | Peptides | Sequence coverage [%] | Score |
| --- | --- | --- | --- | --- | --- | --- |
| 0.00019 | 310.09 | 74873244 | Putative defense protein Hdd11 (*Hyphantria* differentially displayed gene 11) | 9 | 60.7 | 323.31 |
| 1.3E-05 | 87.75 | 186703381 | Hemolin | 7 | 50.7 | 323.31 |
| 6.37E-05 | 80.31 | 112984054 | Yellow-d | 18 | 44.7 | 323.31 |
| 5.38E-05 | 54.42 | 156630481 | Cecropin-D-like peptide | 2 | 14.5 | 46.071 |
| 7.84E-05 | 48.11 | 55139149 | Hemolin (fragment) | 18 | 58 | 323.31 |
| 0.001611 | 42.22 | 112983052 | Chemosensory protein 7; Chemosensory protein11 | 7 | 27.7 | 169.29 |
| 3.6E-05 | 41.13 | 154240658 | Peptidoglycan recognition protein-D | 7 | 22 | 120.85 |
| 2.05E-05 | 26.60 | 183979237 | Transgelin | 6 | 21.1 | 169.94 |
| 0.016753 | 25.55 | 23208535 | Peptidoglycan recognition-like protein B (fragment) | 13 | 34 | 231.94 |
| 0.01404 | 24.64 | 56718390 | Prophenol oxidase activating enzyme 3 | 17 | 42.1 | 210.04 |
| 0.0047 | 23.40 | 110347843 | Protease inhibitor-like protein | 2 | 40 | 31.579 |
| 0.002981 | 20.92 | 45594226 | Serpin-4B | 4 | 70 | 68.718 |
| 0.000752 | 18.60 | 157927723 | Beta actin | 11 | 54.1 | 277.07 |
| 0.0039 | 16.29 | 116087 | Cecropin-A (Cecropin-C) | 1 | 8.5 | 9.783 |
| 0.000134 | 16.07 | 158962507 | Chemosensory protein | 6 | 23.3 | 93.297 |
| 0.02326 | 15.23 | 158962511 | Chemosensory protein | 5 | 16.7 | 110.2 |
| 0.003987 | 15.10 | 226342884 | Serpin-11 | 13 | 39.2 | 166.87 |
| 0.016175 | 14.34 | 23208546 | Heat shock-like protein (fragment) | 6 | 25.6 | 52.216 |
| 0.008751 | 14.00 | 25989211 | CLIP domain-containing serine protease 2 (EC 3.4.21.-) (BzArgOEtase) (BAEEase) [Cleaved into: CLIP domain-containing serine protease 2 light chain; CLIP domain-containing serine protease 2 heavy chain] | 7 | 53.6 | 102.38 |
| 0.005683 | 11.01 | 33860163 | Inducible metalloproteinase inhibitor protein [cleaved into: IMPI alpha] | 8 | 33.2 | 67.368 |
| 0.015931 | 10.84 | 4090964 | Immune-related Hdd1 | 9 | 14.4 | 102.23 |
| 0.007629 | 10.67 | 112702923 | Peptidoglycan-recognition protein-LB (fragment) | 2 | 60.5 | 41.555 |
| 0.000281 | 10.67 | 112983994 | Peptidoglycan recognition protein | 13 | 57.9 | 306.89 |
| 0.017641 | 10.31 | 156254836 | Serpin-2 | 8 | 23.6 | 91.985 |
| 0.000207 | 10.02 | 157704337 | Hdd1-like protein | 6 | 56.9 | 146.94 |
| 0.028818 | 7.68 | 148298796 | Thioredoxin | 5 | 21.8 | 48.041 |
| 0.007352 | 7.61 | 110649240 | Gloverin | 2 | 28.8 | 18.817 |
| 0.001212 | 7.40 | 112984088 | Yellow-fb | 7 | 23.7 | 84.78 |
| 0.013799 | 7.09 | 110347794 | Gloverin-like protein | 5 | 23.2 | 102.62 |
| 0.038579 | 7.07 | 189181680 | Carboxylic ester hydrolase (EC 3.1.1.) | 8 | 49.1 | 138.97 |
| 0.005899 | 6.49 | 110826028 | Esterase | 3 | 8.1 | 32.225 |
| 0.008522 | 5.61 | 112983342 | Tubulin beta chain | 7 | 17.3 | 48.989 |
| 0.024206 | 4.91 | 112983414 | Heat shock protein Hsp21.4 | 5 | 15 | 80.094 |
| 0.019645 | 4.11 | 45594226 | serpin-4B | 3 | 36 | 26.047 |
| 0.014155 | 4.06 | 170578347 | Immunoglobulin I-set domain containing protein | 2 | 35.2 | 14.72 |
| 0.01201 | 4.00 | 39579195 | Odorant-binding protein (pheromone binding protein 4) | 3 | 8.9 | 21.382 |
| 0.000321 | 3.49 | 219552448 | Arginine kinase (Fragment) | 8 | 62.5 | 201.49 |
| 0.000429 | 3.40 | 158289805 | AGAP010733-PA (Fragment) | 12 | 38.1 | 130.56 |
| 0.000956 | 3.40 | 39579195 | Odorant-binding protein | 5 | 19.5 | 41.671 |
| 0.004217 | 2.97 | 73671225 | Superoxide dismutase [Cu-Zn] (EC 1.15.1.1) | 3 | 57.8 | 312.25 |
| 0.000545 | 2.93 | 156968291 | Uncharacterized protein (fragment) | 16 | 35.2 | 244.53 |
| 0.004998 | 2.72 | 162462783 | Carboxylic ester hydrolase (EC 3.1.1.-) | 17 | 30.4 | 323.31 |
| 0.013369 | 2.68 | 220902980 | Carboxylic ester hydrolase (EC 3.1.1.-) | 6 | 36.2 | 158.78 |
| 0.007705 | 2.57 | 27733415 | Serpin 3a (Serpin 3b) | 11 | 12.8 | 243.76 |
| 4.6E-05 | 2.55 | 189181680 | Integument esterase 2 precursor | 19 | 49.1 | 323.31 |
| 0.004825 | 2.47 | 52782700 | Beta-1,3-glucan-binding protein (BGBP) (Beta-1,3-glucan recognition protein) (BetaGRP) (Gram negative bacteria-binding protein) (fragment) | 2 | 14.5 | 39.877 |
| 0.001052 | 2.45 | 15146043 | Alpha-crystallin | 8 | 30.9 | 136.8 |
| 0.013933 | 2.09 | 225031010 | Tubulin alpha chain (fragment) | 3 | 46.2 | 26.541 |
| 0.006553 | 2.02 | 162462783 | Heat shock protein 25.4 | 6 | 12 | 51.116 |
| 0.04006 | 1.62 | 169646838 | heat shock protein 25.4 precursor | 13 | 48 | 237.16 |
| 0.02193 | 1.59 | 239938036 | Beta-galactosidase (EC 3.2.1.23) | 20 | 33.5 | 323.31 |

**Supplementary Table 6: Proteins which are decreased in abundance in *G. mellonella* larvae hemolymph infected with BCG *lux* 1x10^7^ CFU (10 μl inoculum) at 168 h as compared to 0 h uninfected control larvae.**

| p value | Fold change (-) | GI Number | Protein name | Peptides | Sequence coverage [%] | Score |
| --- | --- | --- | --- | --- | --- | --- |
| 0.013035 | 47.43 | 158515746 | Juvenile hormone binding protein | 11 | 52.1 | 227.09 |
| 0.003962 | 29.23 | 229002332 | Hypothetical protein | 3 | 14.5 | 114.37 |
| 0.021672 | 20.83 | 9087198 | Fibrohexamerin (25 kDa silk glycoprotein) (p25) | 13 | 18.1 | 245.2 |
| 0.000215 | 11.23 | 164459610 | Putative hydroxypyruvate isomerase (EC 5.3.1.22) | 13 | 33.3 | 109.06 |
| 0.045961 | 9.58 | 46396271 | 27 kDa hemolymph protein | 8 | 24.7 | 78.91 |
| 0.002178 | 9.14 | 121308868 | Serine proteinase | 9 | 25.4 | 96.748 |
| 0.005229 | 8.52 | 183979239 | Similar to CG10638-PA | 4 | 28.8 | 145.1 |
| 0.000253 | 7.70 | 112983816 | Glyceraldehyde-3-phosphate dehydrogenase (EC 1.2.1.12) | 8 | 38.8 | 67.248 |
| 0.000958 | 6.98 | 116791778 | Uncharacterized protein | 22 | 51.8 | 323.31 |
| 0.000725 | 4.60 | 46396271 | 27 kDa hemolymph protein (P27K) | 13 | 59.6 | 323.31 |
| 0.007426 | 3.96 | 77415676 | Uncharacterized protein | 15 | 32.2 | 323.31 |
| 0.012093 | 3.82 | 114052174 | Aminoacylase | 6 | 17.8 | 52.484 |
| 0.002862 | 3.76 | 18140733 | Twelve cysteine protein 1 | 9 | 43.4 | 163.18 |
| 0.017969 | 3.42 | 182509208 | Carboxylic ester hydrolase (EC 3.1.1.-) | 11 | 39.8 | 86.532 |
| 0.001288 | 3.41 | 114051866 | Isocitrate dehydrogenase [NADP] (EC 1.1.1.42) | 12 | 29.7 | 111.81 |
| 0.000103 | 2.98 | 158515746 | Juvenile hormone binding protein | 15 | 41.7 | 323.31 |
| 0.016866 | 2.90 | 114051325 | Phosphoribosylaminoimidazole carboxylase | 6 | 14.3 | 43.745 |
| 0.022998 | 2.68 | 156630460 | Anionic antimicrobial peptide 2 | 8 | 32.9 | 323.31 |
| 0.010865 | 2.67 | 134103857 | Cationic peptide CP8 | 6 | 29.2 | 323.31 |
| 0.035097 | 2.57 | 153791847 | Nucleoside diphosphate kinase (EC 2.7.4.6) | 10 | 51.7 | 67.351 |
| 0.019017 | 2.49 | 56462286 | Putative protease inhibitor 4 | 6 | 23.2 | 137.05 |
| 0.003218 | 2.44 | 229002332 | Hypothetical protein | 5 | 22.1 | 106.19 |
| 0.003107 | 1.90 | 153791206 | Scolexin | 15 | 28.2 | 323.31 |
| 0.04044 | 1.89 | 170035237 | Aliphatic nitrilase (putative) | 13 | 42.3 | 234.5 |
| 0.002963 | 1.87 | 114050871 | Carboxylesterase | 24 | 62 | 323.31 |
| 0.010259 | 1.79 | 116782397 | Uncharacterized protein | 13 | 37.4 | 323.31 |
| 0.008458 | 1.78 | 237874122 | Multicystatin and procathepsin F precursor | 7 | 48.3 | 168.16 |
| 0.010043 | 1.75 | 70609810 | Transferrin | 58 | 55.1 | 323.31 |
| 0.024724 | 1.75 | 229002332 | Hypothetical protein | 8 | 52.2 | 122.75 |
| 0.034435 | 1.75 | 112983070 | BCP inhibitor | 6 | 21 | 323.31 |
| 0.046289 | 1.74 | 34921426 | Serine protease inhibitor dipetalogastin (Dipetalin) (fragment) | 13 | 28.8 | 323.31 |
| 0.00101 | 1.68 | 2498144 | Apolipophorins [cleaved into: Apolipophorin-2 (Apolipophorin II) (apoLp-2); Apolipophorin-1 (Apolipophorin I) (apoLp-1)] | 12 | 85.9 | 323.31 |
| 0.006845 | 1.61 | 183979392 | Uncharacterized protein | 29 | 56.3 | 323.31 |
| 0.001263 | 1.57 | 229002332 | Uncharacterized protein | 9 | 42.4 | 175.39 |
| 0.046218 | 1.51 | 116245495 | General odorant-binding protein 68 | 12 | 44.1 | 172.16 |

**Supplementary Table 7: BCG *lux* proteins detected in hemolymph over time during infection of *G. mellonella* larvae.**

| LFQ Intensity Value | | | | Uniprot Identifier | Protein Name |
| --- | --- | --- | --- | --- | --- |
| 0 h | 4 h BCG | 48 h BCG | 168 h BCG |  |  |
| 0 | 0 | 2.96E+08 | 0 | A0A0H3MDY6 | Diacylglycerol O-acyltransferase |
| 0 | 0 | 8.89E+07 | 0 | A0A0H3MBF9 | Uncharacterized protein |
| 0 | 0 | 8.04E+07 | 0 | A0A0H3M719 | Possible molybdopterin biosynthesis protein MoeW |
| 0 | 0 | 2.31E+07 | 0 | A0A0H3M8U8 | Probable acyl-CoA dehydrogenase FadE23 |
| 0 | 0 | 1.33E+07 | 0 | A1KPT4 | Lysine--tRNA ligase |
| 0 | 0 | 9.24E+06 | 0 | A0A0H3MB41 | Uncharacterized protein |
| 0 | 6.46E+08 | 0 | 0 | A0A0H3M2K9 | Ribonuclease VapC (RNase VapC) (Toxin VapC) |
| 0 | 3.98E+07 | 0 | 0 | A0A0H3M9E6 | Possible cell division transmembrane protein FtsK |
| 0 | 2.94E+07 | 0 | 0 | A1KFW3 | 2,3-bisphosphoglycerate-dependent phosphoglycerate mutase |
| 0 | 1.56E+07 | 0 | 0 | A0A0H3M591 | Heat shock protein HspX |
| 0 | 1.05E+07 | 0 | 0 | A0A0H3MJ06 | Diacylglycerol O-acyltransferase |
| 0 | 8.85E+06 | 0 | 0 | A1KNQ0 | Adenosylhomocysteinase |
| 0 | 5.96E+06 | 0 | 0 | A0A0H3MEF1 | Possible transmembrane protein |
| 0 | 0 | 0 | 3.07E+09 | A0A0G2Q9J3 | DUF4143 domain-containing protein |
|  |  |  | 3.48E+08 | A0A0H3M2D4 | Possible conserved transmembrane protein |
